# Aleutian disease: Risk factors and ImmunAD strategy for genetic improvement of tolerance in American mink (*Neogale vison*)

**DOI:** 10.1371/journal.pone.0306135

**Published:** 2024-07-18

**Authors:** Seyed Milad Vahedi, Siavash Salek Ardestani, Mohammad Hossein Banabazi, K. Fraser Clark

**Affiliations:** 1 Department of Animal Science and Aquaculture, Dalhousie University, Bible Hill, NS, Canada; 2 Department of Animal Science, University of Zanjan, Zanjan, Zanjan, Iran; 3 Centre for Veterinary Medicine and Animal Science (VHC), Department of Animal Biosciences (HBIO), Swedish University of Agricultural Sciences (SLU), Uppsala, Sweden; 4 Department of Biotechnology, Animal Science Research Institute of IRAN (ASRI), Agricultural Research, Education & Extension Organization (AREEO), Karaj, Iran; Universidad de la Republica Uruguay: Facultad de Ingeniería, URUGUAY

## Abstract

Aleutian disease (AD) is a devastating infectious disease in American mink (*Neogale vison*) industry caused by Aleutian mink disease virus (AMDV). Two crucial steps toward controlling infectious diseases in farm animals are: (i) assessment of the infection risk factors to minimize the likelihood of infection and (ii) selection of animals with superior immune responses against pathogens to build tolerant farms. This study aimed to investigate AD risk factors and evaluate a novel “ImmunAD” approach for genetic improvement of AD tolerance. Phenotypic records and pedigree information of 1,366 and 24,633 animals were included in this study. The risk of animal’s age, sex, color type, and year of sampling on AMDV infection was assessed using a logistic regression model and counter immune-electrophoresis (CIEP) test results. ImmunAD phenotype was calculated based on AMDVG enzyme-linked immunosorbent assay (ELISA) and CIEP test results, and breeding values for ImmunAD were estimated using an animal model. Animals were classified into high-coordinated (HCIR), average-coordinated (ACIR), and low-coordinated immune responders (LCIR) using ImmunAD’s breeding values, and the impact of selection of HCIR on live grade of pelt quality (PQ), harvest weight (HW), and harvest length (HL) breeding values were evaluated. Age of > 1 year, male sex, and year of sampling were identified as significant risk factors of AD (p < 0.05). A moderate-to-high heritability (0.55±0.07) was estimated for ImmunAD, while a higher heritability was observed among the CIEP-positive animals (0.76±0.06). Significantly higher breeding values were observed for PQ and HL among HCIR than those for LCIR and ACIR (p < 0.05). Our findings indicate the critical role of male breeders in AD distribution within mink farms. Regular screening of AD in male breeders before pairing them with females during breeding seasons can help disease control. ImmunAD strategy can be applied to genetic improvement of AD tolerance, with favorable impacts on some growth and production traits. Higher genetic gains can be achieved in populations with higher AD seroprevalences.

## 1. Background

Aleutian disease (AD), or mink plasmacytosis, caused by Aleutian mink disease virus (AMDV), is one of the most severe and costly issues affecting the American mink (*Neogale vison*) breeding industry [1, 2]. Aleutian disease reduces pelt quality and lowers the fertility rate in adult animals [3, 4]. Adults typically develop a chronic form of AD, in which the infection results in progressive wasting syndrome with severe weakness and loss of muscle and fat tissues [1, 5]. The disease can result in a high mortality rate in kits, mainly due to acute interstitial pneumonia [1, 5].

Aleutian disease outbreaks have imposed substantial economic losses on the mink farmers [6–8]. Eradication programs using counter-immunoelectrophoresis (CIEP), the gold standard diagnostic test, could not achieve satisfying results in AMDV eradication programs, which might be due to the high concentration of mink ranches, high seroprevalence of AD among wild mammals, and relatively low sensitivity of the CIEP test [7, 9, 10]. Efforts to find an effective vaccine or a practical treatment were unsuccessful. Therefore, selection for AD tolerance based on quantitative tests measuring antiviral antibodies against viral VP2 capsid protein, e.g., VP2 enzyme-linked immunosorbent assays (ELISA), or those against AMDVG viral antigen, e.g., AMDVG ELISA, has become the priority of mink farmers in some mink breeding countries to build AD-tolerant herds [11, 12].

Two essential strategies to control infectious diseases in farm animals are minimizing the likelihood of infection and selecting animals with superior immune responses against pathogens. Several factors can contribute to the risk of AMDV infection in mink, including the animal’s age, sex, and color type [13–15]. Seroprevalence of AD increases with age [13, 14], suggesting that animals kept on the farm for a longer time have a higher chance of infection. Breeder animals are usually kept longer in mink farming practices, and males are commonly paired with multiple females in breeding seasons, but juveniles are removed from the farm for pelting before reaching the age of one year. Considering the longer time spent on the farm and the interaction with multiple animals, breeder animals might have a higher risk of infection and play an essential role in the distribution of the disease. Some studies found that the animal’s sex can significantly impact the anti-AMDV antibody levels [14, 16, 17]; however, some did not find any significant differences between sexes [13]. Therefore, the impact of sex as an AD risk factor needs to be more investigated. Studies on different color types of mink found various responses against AMDV, but these studies are limited to a few color types [16, 17]. Samples obtained from a specific farm in different years demonstrated significantly different antibody levels [16, 17], suggesting that the average anti-AMDV antibody levels can vary over time due to outbreaks or inconsistency in biosecurity measures.

Logistic regression models have been widely used to investigate disease risk factors in livestock species as it allows for the prediction of the probability of a binary outcome (e.g., presence or absence of a disease) based on one or more predictor variables, making it suitable for identifying which factors contribute to disease occurrence [18–20]. The output is interpretable in terms of odds ratios (OR), making it valuable for understanding the relative impact of different risk factors on disease outcomes [18]. By examining large datasets of serological tests and demographic records of mink populations, logistic regression can identify significant predictors of AD susceptibility. This understanding can then inform targeted preventive measures, such as vaccination strategies, quarantine protocols, or breeding practices aimed at reducing disease transmission and prevalence among mink populations.

The animal’s immune response is a critical factor in determining the final phenotype of AD. Animals with a more robust immune response typically show low disease incidence [21–23]. For instance, mastitis, metritis, ketosis, displaced abomasum, and retained fetal membrane are less frequent among Holstein cattle with high cellular and antibody immune responses [22, 23]. The reason is that the immune system controls an animal’s ability to respond against invading pathogens, while a large portion of the immune response is regulated by the animal’s genetics [24]. Given that cell-mediated immune response is more noticeable in protection against intracellular pathogens such as viruses, AD is more tolerable in animals with higher T cell activities [25, 26]. Proper T helper type 1 and cytotoxic T cell responses against AMDV result in mink’s “non-progressive” form of AD [25, 27]. In this form, animals develop lower levels of anti-AMDV antibody production [25, 27], as they have higher levels of coordination between cell-mediated and antibody immune responses (for more details, see [1]). Therefore, low anti-AMDV antibody levels can be used as a hallmark of high levels of coordination between cell- and antibody-mediated immune responses against AMDV and as a result, AD tolerance. This hallmark can be applied to developing a method to evaluate animals’ genetic merit for the proper immune response against AMDV in the mink industry.

This study used collected records from a Canadian mink farm to identify AD risk factors. We explained and examined a practical method of “ImmunAD” to genetically select animals for high levels of coordination between cell- and antibody-mediated immune responses against AMDV. We provided genetic parameter estimates for ImmunAD and showed the impact of selection for ImmunAD on some production and growth traits.

## 2. Methods

### 2.1 Ethical statement

All procedures applied in this study were approved by the Dalhousie University Animal Care and Use Committee (certification nos. 2018–009, and 2019–012), and mink used were cared for according to the Code of Practice for the Care and Handling of Farmed Mink guidelines [28]. The study is reported in compliance with the ARRIVE guidelines.

### 2.2 Animals and phenotypes

We used the pedigree and phenotypic data previously collected by Miar lab. The phenotypic records belonged to 1,366 American mink from the Canadian Centre for Fur Animal Research (CCFAR) farm at the Faculty of Agriculture of Dalhousie University (Bible Hill, Canada). Animals were kept under standard farming conditions, fed identical diets, and had *ad libitum* access to diet and water. Phenotypic selection based on production traits, particularly pelt quality and reproductive performances, was the primary selection strategy in the CCFAR herd [17]. The CCFAR farm has likely experienced several AD outbreaks, most recently in 2012 and 2013 [7, 12]. Animals were born in 2018 and 2019 by mating 176 sires and 380 dams. The pedigree data included 24,633 animals tracing back to 16 generations.

The harvest length (HL) and harvest weight (HW) were measured in December 2018 and 2019 for animals in their first year at the age of week 31. However, for sires that completed their breeding tasks and for dams mated but were infertile, the traits were measured in February 2018 and 2019. Pelt quality of live animals (PQ) was also graded in November 2018 and 2019 for mink < 12 months old based on the North American Fur Auctions (NAFA) live animal grading procedure (www.nafa.ca). The overall pelt quality scores were given by a skilled technician from NAFA into three categories: 1 (poor), 2 (average), and 3 (best).

For AD assessment, two rounds of blood samples were taken in 2018 and 2019: (i) from all animals, including breeders, in mid-November; (ii) only from breeders in mid-February. At each round, two tests were performed on each animal’s sample: (i) ADMVG ELISA at Middleton Veterinary Services (Middleton, Canada); (ii) CIEP test at Animal Health Laboratory at the University of Guelph (Guelph, Canada). The CIEP test results were reported as positive or negative, representing the presence and absence of anti-AMDV antibodies. In contrast, for AMDVG ELISA, the raw optical densities (OD) were reported without any correction for negative and positive controls, demonstrating the level of anti-AMDV antibodies produced post-infection. The seroprevalence of AD in 2018 and 2019 was obtained using the following formula:

$$\text{AD}\;\text{seroprevalence}\;\left(\%\right)=\frac{\text{No}.\;\text{of}\;\text{CIEP}\;\text{positive}\;\text{individuals}}{\text{Total}\;\text{no}.\;\text{of}\;\text{animals}\;\text{with}\;\text{CIEP}\;\text{record}}\times 100$$

### 2.3 Association study

For association study, animals with repetitive CIEP records (N = 34) were removed; therefore, 1,035 animals with a single CIEP record were included. A logistic regression model was used to evaluate potential risk factors of AD using the *glm* function in R 4.0.5 software [29] as follows:

$${\text{y}}_{\text{ijkl}}=\mu +\alpha \times {\text{A}}_{\text{i}}+{\text{S}}_{\text{j}}+{\text{C}}_{\text{k}}+{\text{Y}}_{\text{l}}+{\text{e}}_{\text{ijkl}}$$

where **y**_**ijkl**_ is the binary CIEP test result (positive and negative), μ is the overall mean, **α** is the regression coefficient**, A**_**i**_ is the animal’s age (> 1 year and < 1 year), **S**_**j**_ is sex (male and female), **C**_**k**_ is color type of animal (dark, demi, mahogany, pastel, and stardust), **Y**_**I**_ is year of sampling (2018 and 2019), and **e**_**ijkl**_ is residual. The associations between the independent and dependent variables were tested using an OR, and ORs with a 95% confidence interval (95% CI) were estimated. An OR value > 1.0 indicated that the group was at higher risk of AD. A chi-square (χ2) test was performed to find statistically significant OR (p < 0.05).

### 2.4 ImmunAD calculation

The ImmunAD approach was implemented in two steps for animals with both AMDVG ELISA and CIEP records (N = 1,103):

1. Log-transformation of AMDVG ELISA OD values to approximate the normality as follows:
ImmunAD=−log(ADMVGELISAOD)
2. Correction of ImmunAD phenotypes for the effect of the presence/absence of AMDV infection using the CIEP test in a univariate animal model.

The reason for multiplication by -1 in the first step is the reverse relationship between the anti-AMDV antibody production level and the fitness of immune response against AMDV [25, 26]. It means that susceptible animals to AD with low coordination between humoral and cellular immune responses produce high levels of anti-AMDV antibodies, whereas, in AD-tolerant individuals, lower levels of anti-AMDV antibodies are expected [26]. In the second step, animals with CIEP-positive records were considered infected, and those with CIEP-negative records were considered AD-free.

### 2.5 Variance components and heritability estimation

To estimate the variance components for ImmunAD, HW, HL, and PQ, a series of univariate animal models were used in Asreml-R 4 [30] as follows:

y=μ+Xb+Za+Wm+Dcl+Epe+e,

where **y** is the vector of observed phenotypic values of the animals, μ is the overall mean population; **b** is the vector of fixed effects (sex, color, year, CIEP test result), **a** is the vector of random additive genetic effects, **m** is the vector of random maternal effects, **cl** is the vector of common litter effects, **pe** is the vector of random permanent environment effects for traits with repeated measures, and **e** is the vector of residual effects. **X,Z,W,** ***D*,** and **E** are the incidence matrices relating the phenotypic observations to fixed, random additive genetic, random maternal, random common litter, and random permanent environment effects, respectively. Random effects of additive genetic, maternal, common litter, and permanent environment were assumed to be normally distributed with means of 0 and variances of ${\text{A}\sigma }_{\text{a}}^{2}{,\text{A}\sigma }_{\text{m}}^{2}{,\text{I}}_{\text{D}}\sigma_{\text{cl}}^{2}{,\text{I}}_{{\text{O}}_{\text{a}}}\sigma_{\text{pe}}^{2}$, and $I_{O_{\text{b}}}\sigma_{e}^{2}$, respectively, where **A** is the genetic relationship matrix, **I**_**D**_ is an identity matrix accounting for the number of dams with offspring, and $I_{O_{\text{a}}}$ and $I_{O_{\text{b}}}$ are identity matrices for the variance of the permanent environment effect and residual effects connecting the records of animals with the observation vector. The significance of fixed effects, including sex (male and female), year (2019 and 2020), and color type (dark, demi, mahogany, pastel, and stardust) were tested for each trait, and only significant ones were retained in the models (p < 0.05). In addition, the results of the CIEP test were implemented in the model as a fixed factor to test the effect of presence/absence of AMDV infection on ImmunAD. The significance of random maternal, common litter, and permanent environment effects was also determined by comparing the full and reduced models using the following formulas:

$$-2\left({\text{log}\;\text{L}}_{\text{reduced}\;\text{model}}-{\text{log}\;\text{L}}_{\text{full}\;\text{model}}\right),$$

$$∼X_{df\left(full\;model\right)-df\left(reduced\;model\right)}^{2},$$

where **log L** and **df** are log-likelihood and degrees of freedom in each model. Nonsignificant random effects (p > 0.05) were removed from the model for variance component estimations.

To investigate the impact of AD prevalence on ImmunAD heritability, variance components of additive genetic, maternal, and residual were further partitioned into CIEP-positive $\left(\sigma_{{\text{a}}_{\text{CIEP}+}}^{2},\sigma_{m_{\text{CIEP}+}}^{2},\sigma_{{\text{e}}_{\text{CIEP}+}}^{2}\right)$ and -negative $\left(\sigma_{{\text{a}}_{\text{CIEP}-}}^{2},\sigma_{{\text{m}}_{\text{CIEP}-}}^{2},\sigma_{{\text{e}}_{\text{CIEP}-}}^{2}\right)$ subpopulations using *at(CIEP)*:*vm(animal*, *ainv)*, *at(CIEP)*:*vm(dam*, *ainv)*, and *dsum(~ units | CIEP)* functions in Asreml-R, respectively.

Random maternal effect was significant (p < 0.05) for ImmunAD and HW, and random permanent environment and common litter effects were nonsignificant for all traits (p > 0.05). Therefore, phenotypic variance was calculated using $\sigma_{\text{p}}^{2}=\sigma_{\text{a}}^{2}+\sigma_{\text{m}}^{2}+\sigma_{\text{e}}^{2}$ for ImmunAD and HW, and $\sigma_{\text{p}}^{2}{+\sigma }_{\text{a}}^{2}{+\sigma }_{\text{e}}^{2}$ for other traits. Heritability was also calculated by $h^{2}=\frac{\sigma_{a}^{2}}{\sigma_{\text{p}}^{2}}$. To investigate the impact of AD seroprevalence on ImmunAD heritability, its heritability was also estimated for CIEP-positive (${\text{h}}_{\text{CIEP}+}^{2}$) and CIEP-negative ($h_{\text{CIEP}-}^{2}$) subpopulations as follows:

$$h_{CIEP+}^{2}=\frac{\sigma_{a_{CIEP+}}^{2}}{\sigma_{a_{CIEP+}}^{2}+\sigma_{m_{CIEP+}}^{2}+\sigma_{e_{CIEP+}}^{2}},$$

$$h_{CIEP-}^{2}=\frac{\sigma_{a_{CIEP-}}^{2}}{\sigma_{a_{CIEP-}}^{2}+\sigma_{m_{CIEP-}}^{2}+\sigma_{e_{CIEP-}}^{2}},$$

Where $\sigma_{a_{\text{CIEP}+}}^{2}$ and $\sigma_{{\text{a}}_{\text{CIEP-}}}^{2}$ are the additive genetic variance of CIEP positive and CIEP negative subpopulations, respectively, $\sigma_{{\text{m}}_{\text{CIEP+}}}^{2}$ and $\sigma_{{\text{mg}}_{\text{CIEP-}}}^{2}$ are maternal variance of CIEP positive and CIEP negative subpopulations, and $\sigma_{e_{\text{CIEP+}}}^{2}$ and $\sigma_{e_{\text{CIEP-}}}^{2}$ are the residual variance of CIEP positive and CIEP negative subpopulations.

### 2.6 ImmunAD classification and association test

We classified animals based on their genetic merit of ImmunAD. First, nine animals with seroconversion from 2018 to 2019 were excluded. Then, the remaining individuals were classified based on the estimated breeding values (EBV) for ImmunAD, as previously described [23, 31]. A measure of one standard deviation (**SD**_**EBV**_) from the mean (**μ**_**EBV**_) of breeding values estimated for ImmunAD (**EBV**_**ImmunAD**_) was considered as a cut-off for animals’ classification. Therefore, mink with ${\text{EBV}}_{\text{ImmunAD}}>\left(\mu_{\text{EBV}}{\text{+SD}}_{\text{EBV}}\right)\text{,}\left(\mu_{\text{EBV}}{\text{-SD}}_{\text{EBV}}\right)\leq {\text{EBV}}_{\text{ImmunAD}}\leq \left(\mu_{\text{EBV}}{\text{+SD}}_{\text{EBV}}\right)$ and $EBV_{\text{ImmunAD}}\leq \left(\mu_{EBV}-SD_{EBV}\right)$ were classified into high-coordinated immune responders (HCIR), average-coordinated immune responders (ACIR), and low-coordinated immune responders (LCIR), respectively. Breeding values for other traits were also estimated using the developed univariate models. The significant differences in the means of breeding values estimated for HW, HL, and PQ among LCIR, ACIR, and HCIR were analyzed using a one-way analysis of variance (ANOVA). Fisher’s exact test was used to perform pairwise comparisons between groups, and the test was conducted by the *fisher*.*test* function in R software. The statistically significant threshold was set to p < 0.05 in all tests.

## 3. Results

### 3.1 Aleutian disease risk factors

Table 1 represents the AD seroprevalence with respect to age, sex, color, and year of sampling, along with the significant risk factors for AD. By grouping the animals into two age groups of > and < 1 year old, we could examine the role of breeder animals (> 1 year old) and juveniles (< 1 year old) in AD distribution. We found that AD was more prevalent among animals with more than one year of age (95.165%) compared to juveniles (80.58%), and the age of > 1 year was a significant AD risk factor (OR = 9.56; 95% CI = 3.40–39.97; p = 0.00). We detected higher AD seroprevalence among males (87.25%) than females (75.81%), and male sex was identified as a significant risk factor for AD (OR = 2.40; 95% CI = 1.72–3.38; p = 0.00). Among different colors, the highest and lowest AD seroprevalence was found among stardust (91.67%) and dark (78.19%) color types; however, color was not a significant risk factor (p > 0.05). Lower seroprevalence was observed in 2019 (77.36%) than in 2018 (87.41%), and the year of sampling was identified as a significant factor contributing to the AD seroprevalence (OR = 0.50; 95% CI = 0.33–0.74; p = 0.00).

**Table 1:** Aleutian disease seroprevalence and its association with animal’s sex, color, age, and year of sampling. Odds ratios (OR) with 95% confident interval (95% CI) were estimated for sex, farming interval, color, and year of sampling (Year). Numbers in bold indicate significant association (p < 0.05).

Factor	Category (%)	Seroprevalence (%)	OR	95% CI	p
**Sex**	Female (50.72)	75.81	-	-	-
	Male (49.28)	87.25	2.40	[1.72, 3.38]	**0.00**
**Color**	Dark (34.11)	78.19	-	-	-
	Demi (23.19)	87.50	1.16	[0.69, 1.99]	0.57
	Mahogany (36.33)	80.05	0.87	[0.59, 1.26]	0.46
	Pastel (5.22)	83.33	1.09	[0.51, 2.54]	0.83
	Stardust (1.15)	91.67	1.85	[0.33, 34.66]	0.57
**Age**	< 1 year (94.00)	80.58	-	-	-
	> 1 year (6.00)	95.16	9.56	[3.40, 39.97]	**0.00**
**Year**	2018 (40.68)	87.41	-	-	-
	2019 (59.32)	77.36	0.50	[0.33, 0.74]	**0.00**

### 3.2 ImmunAD phenotype

**Fig 1:**
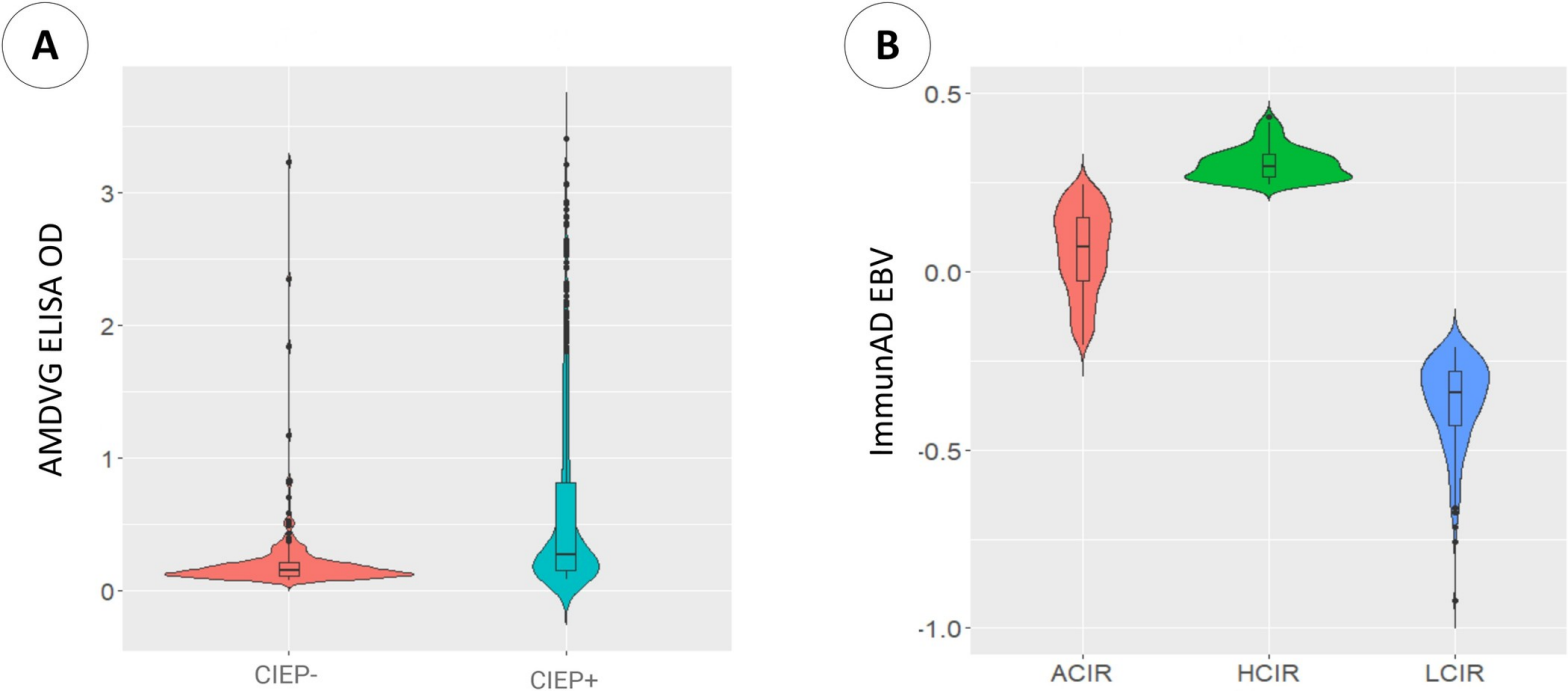
Distribution of AMDVG ELISA records and ImmunAD estimated breeding values (EBVs). Violin plot (A) depicts the distribution of AMDVG ELISA records in two subpopulations of CIEP-negative and -positive. Violin plot (B) shows the distribution of ImmunAD estimated breeding values (EBVs) among high-coordinated (HCIR), average-coordinated (ACIR), and low-coordinated immune responders (LCIR).

The distribution of AMDVG ELISA values across CIEP-positive and -negative animals was shown in Fig 1A, demonstrating a wide range of overlapping OD values between CIEP-negative and -positive animals. Therefore, ImmunAD was calculated based on AMDVG ELISA records corrected for AD incidence using CIEP test results. S1 and S2 Tables provide descriptive statistics and the significance of fixed and random effects on ImmunAD, respectively. ImmunAD values were calculated for 1,103 animals with mean±SD of 0.51±0.42 (S1 Table). The mean of ImmunAD values for females (0.51±0.00) and males (0.50±0.00) were almost comparable, and the sex effect on ImmunAD was found to be nonsignificant (p > 0.05) (S2 Table). Among the color types, the highest mean of ImmunAD belonged to the stardust (0.60±0.03), and the lowest was for the pastel mink (0.31±0.01). However, the color type had a nonsignificant effect on ImmunAD (p > 0.05). The fixed factor of the year was found to be nonsignificant (p > 0.05); however, the mean of ImmunAD in the year 2019 (0.53±0.00) was slightly higher than that of 2018 (0.48±0.00).

### 3.3 Variance components and heritability estimates

Table 2 provides the estimates for variance components and heritabilities of the studied traits. The proportion of ImmunAD variance explained by random permanent environment effects was insignificant (p > 0.05). However, we identified a significant proportion of maternal effect (0.17±0.08) for ImmunAD (p < 0.05). The proportions of variance explained by random maternal and common litter effects were also nonsignificant for HW, HL, and PQ (p > 0.05). High to moderate heritability was obtained for ImmunAD (0.55±0.07) and HL (0.51±0.15), while HW (0.39±0.08) and PQ (0.34±0.07) showed moderate heritabilities.

**Table 2:** Variance components and heritability (h^2^) estimates for studied traits. Additive genetic (V_a_), maternal (V_m_), and residual variance (V_e_) were estimated for ImmunAD, harvest weight (HW), harvest length (HL), and pelt quality grade of live animal (PQ) using univariate models. NE represents values that were not estimated.

Variance components	ImmunAD	HW	HL	PQ
**V**_**a**_	1.40±0.40	0.64±0.22	1.02±0.31	0.17±0.04
**V**_**m**_	0.17±0.08	NE^b^	NE	NE
**V**_**e**_	1.00±0.01	1.00±0.00	1.00±0.32	0.34±0.03
**h** ^**2**^	0.55±0.07	0.39±0.08	0.51±0.15	0.34±0.07

To evaluate the impact of AD seroprevalence on ImmunAD variance components and heritability, we fragmented the population into two groups: (i) animals with CIEP-positive records (N = 902; seroprevalence = 100%) and (ii) animals with CIEP-negative records (N = 201; seroprevalence = 0%). Then, variance components and heritabilities were estimated separately for each subpopulation (Table 3). No ImmunAD variance was explained by genetic variations in the CIEP-negative subpopulation, suggesting that the ImmunAD is not heritable among these animals. However, a remarkably higher additive genetic variance (0.40±0.05) of ImmunAD was estimated for the CIEP-positive subpopulation. Moreover, a higher ImmunAD heritability (0.76±0.06) was estimated for the CIEP-positive subpopulation compared to the CIEP-negative’s (0.00±0.00) and the one for the whole population (0.55±0.07).

**Table 3:** Variance components and heritability (h^2^) estimates of ImmunAD in CIEP-positive and -negative subpopulations recorded for AMDVG ELISA. Additive genetic (V_a_), maternal (V_m_), and residual variance (V_e_) were estimated for ImmunAD in two subpopulations of animals with CIEP-positive (CIEP+) and CIEP-negative (CIEP-) results.

Variance components	ImmunAD	
	CIEP+ (N = 902)	CIEP- (N = 201)
**V**_**a**_	0.40±0.05	0.00±0.00
**V**_**m**_	0.04±0.02	0.00±0.01
**V**_**e**_	0.09±0.02	0.10±0.01
**h** ^**2**^	0.76±0.06	0.00±0.00

### 3.4 Differences between ImmunAD classes

ImmunAD classification was performed for 1,083 animals with ADMVG ELISA test result and single CIEP test record. Using breeding values estimated for ImmunAD, animals were classified as LCIR, ACIR, and HCIR (Fig 1B). As expected, most of the animals (N = 739) were classified as ACIR, and two smaller groups of animals with two extreme genetic merits of ImmunAD, i.e., LCIR (N = 186) and HCIR (N = 158), were identified.

Table 4 represents the results of association tests between ImmunAD classes and breeding values estimated for growth and production traits. Regarding HW, the mean EBV among ImmunAD classes was not significantly different among classes (p > 0.05). However, for HL, we found significantly higher EBVs among HCIR (0.48±1.20) compared to ACIR (0.01±1.33) and LCIR (-0.11±1.36) (p = 0.02). Furthermore, a significantly higher mean of EBV for PQ was observed for HCIR (0.17±0.32) than ACIR (0.08±0.30) and LCIR (0.07±0.32) (p = 0.03).

**Table 4:** Associations between the ImmunAD classes [high-coordinated (HCIR), average-coordinated (ACIR), and low-coordinated immune responders (LCIR)] and the estimated breeding values of harvest weight (HW), harvest length (HL), and pelt quality grade of live animal (PQ) traits. Values indicate the average of estimated breeding values of the trait among individuals in the ImmunAD class. Only animals with HW, HL, and PQ records were included in the association tests. Significant differences in the average of estimated breeding values among the classes of each trait are shown with * and † symbols.

ImmunAD class^a^	HW (p = 0.17)		HL (p = 0.02)		PQ (p = 0.03)	
	N^c^	mean±SD^d^	N	mean±SD	N	mean±SD
**HCIR**	59	0.00±0.11^*^	59	0.48±1.20^*^	95	0.17±0.32^*^
**ACIR**	309	-0.02±0.14^*^	309	0.01±1.33^†^	447	0.08±0.30^†^
**LCIR**	76	-0.04±0.14^*^	76	-0.11±1.36^†^	105	0.07±0.32^†^

^a^ HCIR = high-coordinated immune responders, ACIR = average-coordinated immune responders, LCIR = low-coordinated immune responders.

^b^ CIEP+/- = animals with CIEP positive or negative record, CIEP+ = animals with CIEP positive record, CIEP- = animals with CIEP negative record.

^c^ N = number of animals in the class.

^d^ SD = standard deviation

## 4. Discussion

Aleutian disease has been one of the leading health problems of the mink industry. The lack of adequate means of AD treatment and prevention doubles the importance of effective control strategies in this industry. Here, we focused on two critical aspects of AD control: (i) understanding the risk factors of AD and (ii) genetic selection of animals for AD tolerance using the ImmunAD strategy. While there has been limited study on the AD risk factors, discovering these risk factors can help mink farmers implement preventive measures to minimize the likelihood of AD outbreaks. It also empowers farmers to monitor specific factors closely and take prompt actions, such as quarantine measures or diagnostic testing, in the early stages to prevent AD spread. Several AD tests, such as CIEP and AMDVG ELISA, have been applied in the phenotypic selection of AD-tolerant mink and eradication programs in some mink-producing areas [1, 7, 32]. However, phenotypic selection relies solely on observable traits without considering underlying genetic factors, leading to slower progress in genetic improvement compared to genetic selection methods [33]. It is also prone to environmental influences, resulting in less accurate predictions of offspring performance [34]. More recently, some AD tests were employed in the genetic evaluation of AD tolerance [16, 17], suggesting the importance of a reliable selection method for genetic improvement of this trait in the mink industry. Genetic selection programs have been widely used in animal breeding of different species, such as cattle [33], pigs [35], sheep [36], and horses [37], for many years. Here, we introduced a reliable genetic improvement strategy for proper immune response against AMDV in American mink based on two standard serological tests, CIEP and AMDVG ELISA.

### 4.1 Aleutian disease risk factors

We found that the animals aged > 1 year had a 9.56 times higher risk of AD than younger animals. Our result indicates the significant role of breeder animals in AD spread as these animals are typically kept for more than a year in mink farming practice, but juveniles are removed from the farm for pelting before reaching the age of one year. In parallel to our results, several studies have shown that the seroprevalence of AD in the juvenile mink is lower than in the adult mink [13, 14]. Animals kept in the herd for a longer time are more likely to be exposed to the virus and infected by AMDV. Breeder animals can act as reservoirs in AD-positive farms and transfer the infection to the kits (vertical transmission) and other animals (horizontal transmission) [1, 26]. Therefore, special attention and regular AD measures on breeder animals, such as regular serological tests and quarantine of suspicious animals, should be part of AD control programs in mink farms.

We found that males have a 2.40 times higher risk of AD compared to females. In parallel to our results, some studies found that the animal’s sex can significantly impact the anti-AMDV antibody levels [14, 16, 17]. Considering that a male mink can be paired with multiple females on the mink farm, male animals, particularly male breeders, are more prone to AD, and once infected, they play an important role in the distribution of the virus on the farm. Therefore, conducting regular AD tests on males, especially prior to pairing them with females in the breeding seasons, could be helpful in AD control.

Statistical analysis showed that no color type had a significantly higher risk of AD. In agreement with our results, in a recent study by Virtanen et al. [38], no significant differences was observed in antibody titers of white, sapphire, and brown color types in ¾ of samples. In contrast, Anderson et al. [39] showed that the Hedlund white mink has a significantly higher risk of AD than brown and silver-blue color types. In another studies, Sapphire mink showed higher levels of antibody production post-inoculation than the pastel genotype [15], and sapphire mink are more susceptible than pastel to the Pullman isolate of AMDV [40]. However, none of the above-mentioned color types were included in this study, and this discordance may be due to the different color types applied to our study.

Animals sampled in 2018 had a significantly higher risk of AD. Factors such as disinfection practices, biosecurity measures, and selection strategies could contribute to the different AD seroprevalence in different breeding seasons [41]. Consistency in these practices and measurements can minimize the differences in the risk of disease in different years and the risk of outbreaks.

### 4.2 ImmunAD strategy

We introduced the novel ImmunAD strategy based on two standard serological tests, CIEP and AMDVG ELISA. The CIEP was already used to identify and cull AMDV-positive animals in eradication programs [7, 9], while the AMDVG ELISA was implemented to select AD-tolerant animals in conventional phenotypic selection plans [11]. While multiple high-throughput ELISA platforms have been developed for AD screening in mink farms with sensitivity and specificity of more than 96% and 97% [32, 42], there are some rapid and highly sensitive diagnostic molecular methods, such as polymerase chain reaction (PCR), that can be used for AMDV detection and infection confirmation [43, 44]. The main disadvantage of molecular techniques is that they have high sensitivity and specificity on samples taken from some specific organs, mainly spleen, that are not appropriate for high throughput screening and selection purposes [43, 44]. Meanwhile, ELISA tests can be simply performed on blood samples taken by nail clipping and are a better choice for regular farm screenings and selection strategies [32, 42]. Moreover, molecular techniques can be used to detect AMDV but lack the power to show the animal’s antibody response, which is an essential piece of information for selecting tolerant animals.

Our study indicated that diseased AD-tolerant and healthy animals could obtain comparable OD values in AMDVG ELISA, resulting in low accuracy of conventional selection for AD tolerance. The ImmunAD method transforms the ADMVG ELISA OD into a statistic which is positively correlated with the fitness of immune response against AMDV. Interestingly, we found that when ImmunAD is corrected for presence/absence of AMDV infection using the CIEP test, none of the factors of age, sex, color, and year are significant (S2 Table). Therefore, ImmunAD can efficiently show the AD-tolerant animals using only pedigree information and AMDVG ELISA and CIEP test results. ImmunAD is a much less labor-intensive approach to other genetic improvement strategies that have been already proposed [16, 17] as it does not require collecting the animal’s demographic records such as age, sex, color, and year.

We estimated moderate-to-high heritability (0.55±0.07) for ImmunAD. Our result agrees with Bishop and Woolliams’s theory [45], proposing that traits describing components of immune responses to infection, e.g., antibody production, are often highly heritable. The heritability of antibody response against AMDV using an ELISA platform was previously reported as 0.35±0.06 [17] and 0.26±0.05 [16]; however, different fixed effects and assumptions were considered in their statistical models. In other species, moderate-to-high heritabilities were estimated for antibody response [46–48]. For instance, pigs’ heritability for immunoglobulin G level against porcine reproductive and respiratory syndrome virus was estimated at 0.45±0.13 [46]. Another example is the heritability estimate of 0.16 to 0.59 in European sea bass for antibody titers against the nervous necrosis virus [47]. Recently, a high heritability of 0.42±0.06 was obtained for antibody immune response against clostridial vaccine in Australian Angus cattle [48].

Intriguingly, we observed a higher ImmunAD heritability (0.76±0.06) for the CIEP-positive subpopulation compared to the one estimated for the whole population (0.55±0.07) and CIEP-negative subpopulation (0.00±0.00) (Table 4). The underestimation of ImmunAD heritability among the CIEP-negative subpopulation could result from its low genetic variance (0.00±0.00) compared to CIEP-positive animals (0.40±0.05). However, the small sample size of CIEP-negative subpopulation could be another reason, and further investigation of ImmunAD genetic variation using larger populations is required. In agreement with our results, Bishop and Woolliams [45] showed a positive linear relationship between the prevalence of a viral disease and the heritability of resistance. Moreover, Van Hulzen et al. [49] found that heritability estimations for antibody response against John’s disease are sensitive to within-herd disease prevalence. They revealed that higher heritability is estimated when subsets of the population with a higher incidence of John’s disease are included in the model [49]. Our results indicated that genetic improvement for AD tolerance can be faster in herds with higher AD seroprevalence.

Classification of animals using the breeding values estimated for antibody response has been previously used in genetic evaluation studies [31, 50, 51]. Our classification was based on the breeding values estimated based on two immunoassays of AMDVG ELISA and CIEP; the first was used as phenotypic observation and the latter as a fixed effect in the implemented animal model. We found significantly higher EBVs among HCIR for PQ and HL compared to the ACIR and LCIR. Our results are in agreement with previous studies demonstrated that AMDV might depress pelt market value and animal growth [3, 26]. However, we demonstrated that genetically AD-tolerant animals have higher genetic merits for pelt quality and body length. Therefore, genetic selection for ImmunAD can indirectly improve the herd’s genetic merit for some production and growth traits which is favorable for the mink industry.

## 5. Conclusions

This study was aimed to address: (i) AD risk factors, (ii) introduce and examine the ImmunAD strategy for genetic improvement of AD tolerance, and (iii) estimate its heritability and show the impact of selection for ImmunAD on some production and growth traits. We found age and sex significantly increase the risk of AD, and male breeders play a significant role in AD distribution. Regular AD screening of male breeder animals before pairing them with females should be implemented in AD control. We introduced the novel ImmunAD strategy that may provide a practical genetic evaluation of AD tolerance in American mink. This approach could be the first step toward developing genetic strategies to reduce the economic losses of AD in the mink industry by selecting animals with higher genetic values for the proper immune responses against AMDV. ImmunAD is less labor-intensive than current genetic improvement strategies as it can efficiently show AD-tolerant animals using only three types of records: (i) pedigree information, (ii) AMDVG ELISA, and (iii) CIEP test records. The moderate-to-high heritability of ImmunAD indicated that it could be applied to the mink industry. Genetic improvement can be even faster in herds with higher AD seroprevalence as higher ImmunAD’s heritability was observed in the CIEP-positive subpopulation. Genetic selection for ImmunAD can indirectly improve the herd’s genetic merit for some production and growth traits.

## Supporting information

S1 Table Summary of descriptive statistics for studied traits. Number of samples (N), mean, standard deviation (SD), minimum (Min), and maximum (Max) for ImmunAD, harvest weight (HW), harvest length (HL), and pelt quality grade of live animal (PQ) in animals included in this study.
(DOCX)

S2 Table Description of covariates, fixed effects, and random effects tested in animal models. Covariates, fixed, and random effects used in the univariate models of ImmunAD, harvest weight (HW), harvest length (HL), and pelt quality grade of live animal (PQ). Fixed effects were age, counter-immunoelectrophoresis test result (CIEP), sex, color, and year of sampling (Year). Random effects included maternal (Mat), common litter (ComLit), and permanent environment (PerEnv). For ImmunAD, age means age at the time of sampling, but for HW and HL, means age at the harvest time. NS and NT represent effects that were not significant and those that were not tested, respectively. Significant threshold was considered as p < 0.05.
(DOCX)

## List of abbreviations

ACIR: average-coordinated immune responders
AD: Aleutian disease
AMDV: Aleutian mink disease virus
ANOVA: one-way analysis of variance
CCFAR: Canadian Centre for Fur Animal Research
CIEP: counter-immunoelectrophoresis
EBV: estimated breeding values
ELISA: enzyme-linked immunosorbent assays
HCIR: high-coordinated immune responders
HL: harvest length
HW: harvest weight
LCIR: and low-coordinated immune responders
NAFA: North American Fur Auctions
OD: optical densities
OR: odds ratio
PQ: Pelt quality of live animals
