# Supplementary material for: Aleutian disease: Risk factors and ImmunAD strategy for genetic improvement of tolerance in American mink (Neogale vison)
Source: PLoS One. 2024 Jul 18;19(7):e0306135. doi: 10.1371/journal.pone.0306135 (PMC11257266; doi:10.1371/journal.pone.0306135)
Supplement: S1 Table — Number of samples (N), mean, standard deviation (SD), minimum (Min), and maximum (Max) for ImmunAD, harvest weight (HW), harvest length (HL), and pelt quality grade of live animal (PQ) in animals included in this study. (DOCX) [file pone.0306135.s001.docx]

**S1 Table. Summary of descriptive statistics for studied traits.** Number of samples (N), mean, standard deviation (SD), minimum (Min), and maximum (Max) for ImmunAD, harvest weight (HW), harvest length (HL), and pelt quality grade of live animal (PQ) in animals included in this study.

| Trait | N | Mean | SD | Min | Max |
| --- | --- | --- | --- | --- | --- |
| ImmunAD | 1103 | 0.51 | 0.42 | -0.53 | 1.55 |
| HW | 811 | 2.12 | 0.64 | 0.94 | 3.94 |
| HL | 811 | 44.97 | 4.71 | 33 | 59 |
| PQ | 959 | 1.98 | 0.74 | 1 | 3 |
