# Supplementary material for: Aleutian disease: Risk factors and ImmunAD strategy for genetic improvement of tolerance in American mink (Neogale vison)
Source: PLoS One. 2024 Jul 18;19(7):e0306135. doi: 10.1371/journal.pone.0306135 (PMC11257266; doi:10.1371/journal.pone.0306135)
Supplement: S2 Table — Covariates, fixed, and random effects used in the univariate models of ImmunAD, harvest weight (HW), harvest length (HL), and pelt quality grade of live animal (PQ). Fixed effects were age, counter-immunoelectrophoresis test result (CIEP), sex, color, and year of sampling (Year). Random effects included maternal (Mat), common litter (ComLit), and permanent environment (PerEnv). For ImmunAD, age means age at the time of sampling, but for HW and HL, means age at the harvest time. NS and NT represent effects that were not significant and those that were not tested, respectively. Significant threshold was considered as p < 0.05. (DOCX) [file pone.0306135.s002.docx]

**S2 Table. Description of covariates, fixed effects, and random effects tested in animal models.** Covariates, fixed, and random effects used in the univariate models of ImmunAD, harvest weight (HW), harvest length (HL), and pelt quality grade of live animal (PQ). Fixed effects were age, counter-immunoelectrophoresis test result (CIEP), sex, color, and year of sampling (Year). Random effects included maternal (Mat), common litter (ComLit), and permanent environment (PerEnv). For ImmunAD, age means age at the time of sampling, but for HW and HL, means age at the harvest time. NS and NT represent effects that were not significant and those that were not tested, respectively. Significant threshold was considered as p < 0.05.

| Trait | Covariate | Fixed effects | | | | Random effects | | |
| --- | --- | --- | --- | --- | --- | --- | --- | --- |
|  | **Age^a^** | **CIEP** | **Sex** | **Color** | **Year** | **Mat** | **ComLit** | **PerEnv** |
| ImmunAD | NS^c^ | * | NS | NS | NS | * | NS | NS |
| HW | NS | NT | * | NS | * | NS | NS | NT |
| HL | NS | NT | * | NS | NS | NS | NS | NT |
| PQ | NT | NT | NS | * | * | NS | NS | NT |
